# Exploring the relative influence of raw materials, percussion techniques, and hominin skill levels on the diversity of the early Oldowan assemblages: Insights from the Shungura Formation, Lower Omo Valley, Ethiopia

**DOI:** 10.1371/journal.pone.0283250

**Published:** 2023-04-05

**Authors:** Anne Delagnes, Michel Brenet, Brad Gravina, Frédéric Santos

**Affiliations:** 1 PACEA—De la Préhistoire à l’Actuel: Culture, Environnement et Anthropologie, Université de Bordeaux/CNRS/Ministère de la Culture, Pessac, France; 2 Inrap-Nouvelle-Aquitaine & Outre-Mer, Bègles, France; 3 Musée national de Préhistoire, Les Eyzies-de-Tayac, France; Griffith University, AUSTRALIA

## Abstract

The eastern African Oldowan has been documented in multiple raw material contexts and physical environments and displays considerable differences in terms of technological complexity. The relative influence of percussion techniques and raw material quality are central to debates concerning hominin skill levels as a potential driver of change during the period between 2.6 and 2 million-years (Ma). The early Oldowan assemblages from the Shugura Formation play a key role in these debates due to a number of distinctive features, including the small size of the artefacts and poorly controlled flaking. Here we mobilize quantified and replicable experimental data in order to (a) assess the significance of the bipolar technique in the Omo archaeological assemblages and (b) discriminate the respective impact of raw materials, technical choices and knapper skill levels on the unique character of these assemblages. By combining descriptive statistics with regression tree models, our analysis demonstrates knapper skill level to be of minimal importance in this context for the production of sharp-edged flakes. The absence of a link between skill and knapping success reflects the combined effect of raw material constraints, the frequent use of the bipolar technique, and relatively simple technical objectives. Our analysis confirms the key role played by local environmental conditions in the unique appearance of the Shungura assemblages, a relationship which has been frequently suggested but never demonstrated. Beyond the operational and sensorimotor skills considered in most studies, we suggest that the diversity of early Oldowan assemblages should be better sought in the cognitive abilities developed by early toolmakers as a response to landscape learning and use, two elements of early human evolution that remain largely unexplored.

## Introduction

The Oldowan (ca. 2.6 to 1.7 Ma) lithic assemblages of eastern Africa share a simple yet effective core-flake technology for the production of what are generally unmodified sharp-edged flakes [1–4]. These assemblages nevertheless display some diversity in terms of complexity, the origin of which has long been the matter of debate [2, 3, 5–12]. This issue is particularly relevant for the archaeological record of the Shungura Formation in the Lower Omo Valley, Ethiopia (Member F, 2.32 to 2.23 Ma) [13, 14]. Its distinctive features include the small size of the artefacts and poorly controlled flaking, resulting in high proportions of angular fragments and flake fragments, with correlatively low proportions of complete sharp-edged flakes [15–20]. Diverse causal mechanisms have been advanced to explain the unique appearance of the Shungura assemblages, alternatively invoking environmental factors, such as the small size of the quartz pebbles available in the Lower Omo Valley [16–19, 21], technical choices [18], and/or knapper skill level [22, 23]. Here we mobilize quantified and replicable experimental data in order to (a) assess the significance of the bipolar technique in the Omo archaeological assemblages and (b) discriminate the respective impact of raw materials, technical choices and knapper skill levels on the unique character of these assemblages. In order to overcome limitations inherent in empirical approaches for addressing complex multi-causal issues, we assess the role of each of these three factors both independently and in combination through the use of descriptive statistics combined with regression trees.

Raw material constraints are the main factor held to explain the singularity of the Shungura assemblages [16–20]. All modern knappers agree that quartz is one of the most difficult to work raw material due to its tendency to fracture randomly, following multiple fracture planes in three dimensions from the impact point [24, 25]. This relatively uncontrollable fracturing results in high proportions of angular fragments [17] and “split” flake fragments [16] in the Shungura assemblages. However, the respective impact of quartz on flake fragmentation in relation to other factors remains to be established. In the context of the Lower Omo Valley, the highly variable knapping quality of quartz pebbles must be integrated in such a consideration. The small size of the quartz pebbles available in the Lower Omo Valley, with a mean maximum length of between 3.3 and 4.2 cm (excluding pebbles > 2cm) [26], accounts for the diminutive size of the assemblages, as already pointed by Merrick [19].

The use of bipolar percussion in the Shungura assemblages was first noted by Chavaillon for a core fragment from Omo 123k [15], and equally noted by de la Torre for a chert core from the same site [16]. On the other hand, the predominant if not exclusive use of the bipolar technique was suggested by Ludwig, based on his own experimentally-derived observations and reanalysis of assemblages from Merrick’s excavations (FtJi1, FtJi2, FtJi5 site complexes) [18]. This technical choice was interpreted by the same author as the only possible choice available to knappers in order to split the water-rounded quartz pebbles available in the Lower Omo Valley. Bipolar flaking is strongly linked to the exploitation of quartz at a number of African ESA sites [27–32]. Experimental studies repeatedly highlight the benefits of this technique when applied to quartz, such as facilitating core reduction [28, 33], reducing fragmentation [34], or efficiently producing cutting edges [28, 35, 36]. On the other hand, the relative importance of bipolar percussion with regard to freehand percussion has never been assessed for the Shungura assemblages.

Inferences regarding the degree of technical skill and dexterity of the Shungura toolmakers are even more controversial. The exploitation of minuscule cores and flakes has been interpreted as reflecting an advanced degree of grip precision and manual dexterity [18, 37]. The Shungura assemblages have also been described as simple but efficient and readily adapted to the mediocre quality of the raw material [16, 18, 37]. On the other hand, the prevailing image of an expedient technology limited to "the intensive smashing of small quartz lumps and pebbles", as emphasized by Merrick [19, 20], has led several authors to question the degree of elaboration of the Shungura assemblages [38]. Subsequently, it was suggested that the Shungura assemblages were indistinguishable from those produced by non-human primates [39]. This echoes previous descriptions of the Shungura assemblages as reflecting a “pre-Oldowan stage” characterized by poor technological skills, evident in the lack of control over basic stone knapping principles and poor management of lithic resources [23, 40]. These views fueled an “ape’s view” of the Oldowan as a whole, with these industries seen as a stage of hominin cognitive evolution that shares many of the tool-based practices with extant apes [41, 42]. Although views on the earliest Oldowan assemblages have evolved significantly over the past decades [4], particularly since the stunning evidence of elaborate reduction sequences recovered from Lokalalei 2c [12] and Gona [6], the dominant perception of the Shungura assemblages remains that of poorly elaborated industries.

In sum, each of the factors advanced to explain the unique character of the Shungura assemblages has been the subject of diverging interpretations. These contrasting views are deeply rooted in research paradigms and fostered by a drastic lack of solid analytical data. The diminutive size of the Shungura assemblages is clearly related to the small size of the available pebbles in the Lower Omo Valley, which, combined with the variable quality of the pebbles, required adaptive solutions from the toolmakers. One of the most common solutions is the use of bipolar percussion, extensively documented in quartz assemblages around the world and from all periods of prehistory [29]. However, its role in the Shungura assemblages remains to be determined. Insofar as the impact of each of these factors, raw material, technical choices and knapping skill, has not been clearly established, their respective influence in accounting for the simple technical objectives of the Shungura toolmakers remains impossible to accurately evaluate, a problem compounded by the fact that these factors likely interacted to some extent. Here we present an alternative quantitative approach based on experimental data designed to identify interactions between these different factors and thus provide new insights for discussing the mechanisms contributing to the unique appearance of the Shungura assemblages and the diversity of the Oldowan techno-complex more generally.

## Materials and methods

### Knapper skill levels, raw material quality, and technical choices

The question of knapper skill level is addressed here based on experimental reduction sequences produced by modern knappers with highly contrasting experience. “Skill” is a generic term that encompasses a large variety of distinct notions. For the present analysis, we make a distinction between (a) cognitive skills, i.e. knowledge, (b) operational skills, i.e. know-how, and (c) sensorimotor skills, including precision grip, striking accuracy, and control of percussive force [43–45]. We focus on operational and sensorimotor skills, grouped together under the generic term of “technical skill”, and exclude notions of knapper cognition which cannot be easily addressed through experiments carried out by modern knappers.

**Fig 1:**
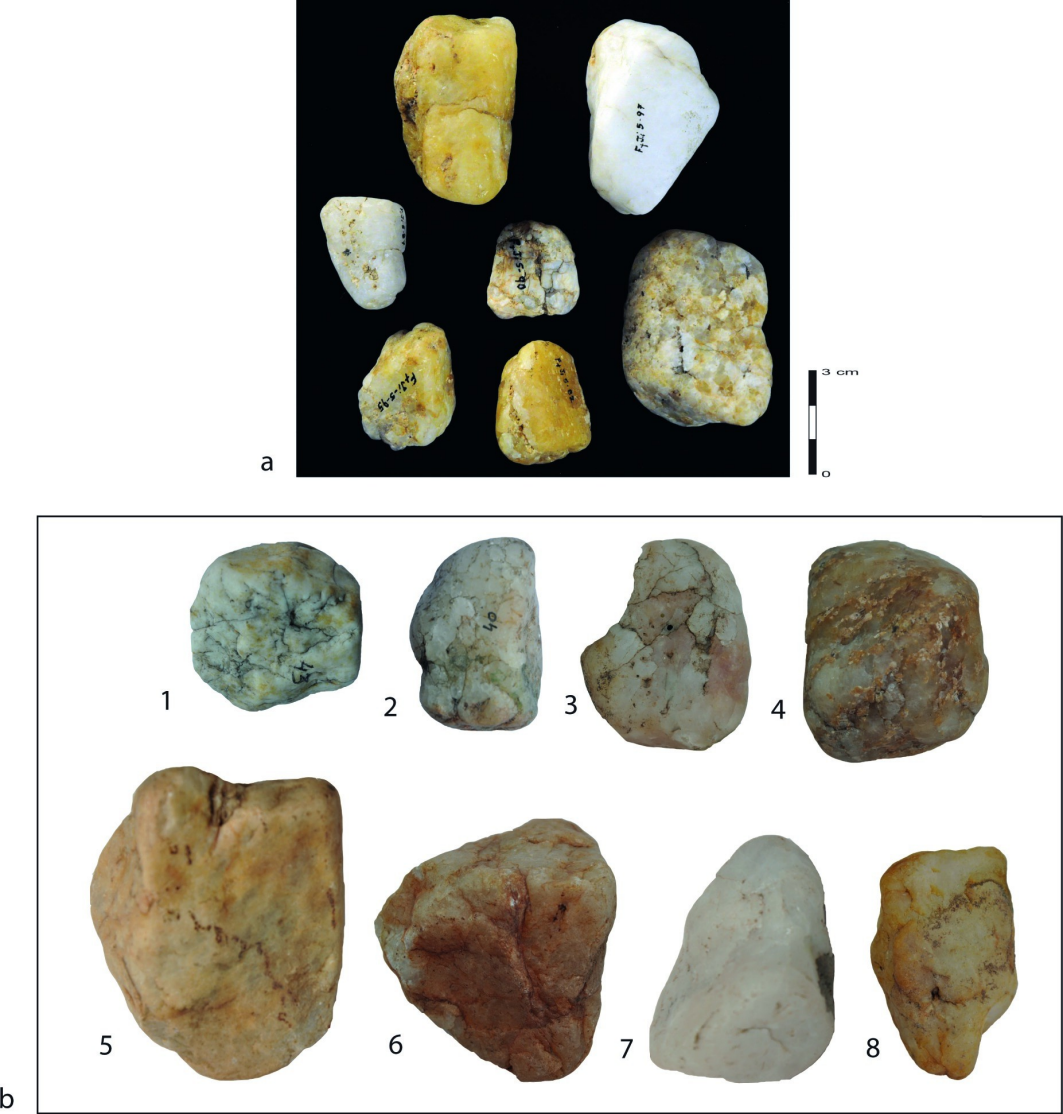
**a. Selection of archaeological quartz pebbles from FtJi5 site complex** [17, 20]. This site complex is found in a conglomerate at the bottom of Member F and provides a unique abundance of unmodified quartz pebbles in archaeological context at the Shungura formation scale; **b. experimental quartz pebbles from the Lower Omo Valley.** 1 to 4: medium to low quality quartz pebbles due to internal cracks (1), coarse grain structure (2 and 3), or heterogeneous matrix (4), 5 to 8: good quality quartz pebbles. NB. Scale identical to **a**.

The majority of raw materials exploited by the Shungura toolmakers are vein quartz pebbles [26], i.e. clasts < 64 mm in length, that derive from the Precambrian basement of the Hamar Range (Fig 1A), the closest secondary sources of which are currently about 15 km east of the main archaeological site complexes. Its petrographic diversity includes quartz, quartz-rich gneiss and quartz feldspar, gathered here under the generic term “quartz”. For the purposes of this analysis, we focus uniquely on the knapping quality of the pebbles, designated as good or medium to low. Good quality quartz pebbles correspond to homogeneous, usually fine-grained quartz that permits relatively good control over the detachment of flakes. Medium to low quality quartz pebbles are more often coarse-grained, and/or with internal cleavage planes or inclusions that make the detachment of flakes considerably more difficult to control. Both groups are present in the experimental material (Fig 1B). Knapping quality of each pebble was recorded before each experimental reduction sequence and, if erroneous (i.e. appearance of cleavage plans, coarse rather than fine-grained matrix), was corrected at the end of the experiment.

**Fig 2:**
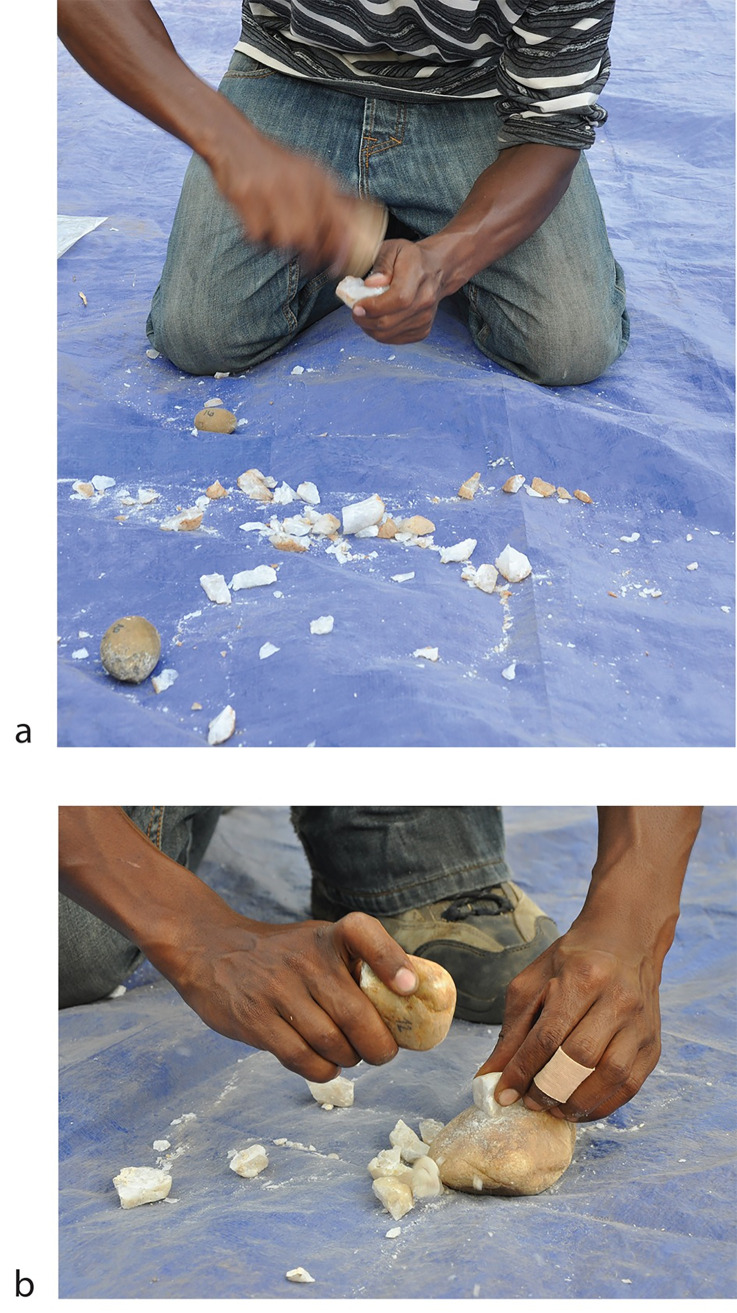
a. Free-hand percussion technique performed by a novice knapper; b. Bipolar technique performed by a novice knapper.

Two technical choices are considered in our analysis: free-hand and bipolar knapping, two percussion techniques implying radically distinct motions and implements (Fig 2). The free-hand technique involves a hand-held mobile hammerstone used to strike a core held in the other hand, with platform angle < 80° (Fig 2A). Bipolar knapping involves a mobile hammerstone and a passive stone or anvil. The core is placed on the anvil and struck vertically, or with a slightly oblique blow, from an opposite platform [46] forming an angle of up to 90° (Fig 2B). The counterblow on the extremity in contact with the anvil produces impact damage (e.g. crushing, cracks, split fractures…) similar to those that develop on the opposite platform. The most diagnostic features, which are exclusive to this technique, are thus cores and flakes with opposed impact scars, developed unifacially or multifacially. Diagnostic bipolar cores are rare, as in their final stage of reduction cores are most often indistinguishable from angular fragments, in particular in contexts involving quartz and where cores have been reduced until exhaustion. Diagnostic flakes are also rare, limited to invasive flakes which show diagnostic impact features on the edge in contact with the anvil as well as on the opposed striking platform. All other features described from experimental studies (e.g. a cubic core shape, unidirectional removals, shattered platforms or bulb shearing, high proportions of angular fragments) are not exclusive to the bipolar technique and therefore cannot be directly extrapolated to archaeological assemblages for identifying bipolar products. Other elements that can be diagnostic of percussion technique, such as the shape of the bulb area [35], are not relevant for the Shungura quartz flakes which almost never present developed bulbs with Hertzian cones. Furthermore, most experimental studies show that discriminating both techniques in quartz assemblages is highly challenging [47–51], and the Shungura assemblages are no exception. However, assessing the relative significance of the bipolar technique in the Shungura assemblages appears essential for testing its relevance as a factor that might have potentially impacted knapping success.

### Selected variables

The Shungura lithic assemblages are the result of the reduction until exhaustion of small unprepared quartz pebbles for the production of sharp-edged flakes that did not require modification prior to use. This functional requirement, which is common to all early Oldowan assemblages, underlies the choice of variables included in the analysis. These variables relate exclusively on the absence / presence and extension of cutting edges, which we consider as the most relevant proxies for assessing the success of the knapping sequence. Our analysis takes into account three independent variables: proportion of angular fragments, proportion of sharp-edged flakes and extension of the cutting edges on the sharp-edged flakes. Angular fragments are by-products devoid of sharp (i.e. cutting) edges, lack a platform, and bear no identifiable ventral or dorsal surface indicative of a controlled detachment. Sharp-edged products include all flakes and broken flakes with effective cutting edges for penetrating soft tissue, defined here as an edge angle ≤ 70°, and maximum length > 2 cm. The proportions of angular fragments and sharp-edged products are calculated in relation to the total number of products extracted from each pebble, and are therefore unaffected by variations in pebble size. The extension of the cutting edges is calculated as the ratio cutting edge length (mm) to the overall circumference of the piece (mm). This ratio is preferred to other values, such as the length of cutting edge alone [18] or flake edge (cm) / flake mass (g) [52, 53], which can be impacted by other variables, including raw material size or matrix.

### Archaeological assemblages

The presence and significance of the bipolar technique is estimated in five archaeological assemblages from Member F, dated to between 2.324 ± 0.020 and 2.271 +/- 0.041 Ma [13, 14]. These assemblages, namely Ftji2 –OMO A2 (abbreviated as FtJi2 –A2, formerly referred to as FtJi2), which combines the names of site complex and archaeological occurrence, OMO 123 –A13 (formerly Omo 123k), and OMO 79—A43, OMO 79 –A82, OMO 371N –A167 were chosen because of their richness (Table 1) and good degree of assemblage integrity in relation to the preservation of artefacts in primary *in situ* position. The first two assemblages derive from excavations carried out in the 1970s by the IORE team [15, 19, 20], completed by a smaller portion of the OMO 123 –A13 assemblage recovered from a test trench and surface surveys as part of the OGRE team in 2008. The last three assemblages come from excavations and test trenches carried out as part of the OGRE in 2018 at OMO 79 and in 2019 at OMO 371N, both of which being newly documented site complexes. The significance of the bipolar technique in the archaeological assemblages was assessed by comparing the proportions of bipolar diagnostic flakes in the archaeological and experimental assemblages. After an omnibus Fisher exact test comparing the proportions of diagnostic flakes among all assemblages, five additional Fisher exact tests were performed as a post-hoc analysis. Each of these tests includes only the experimental assemblage and one given archaeological assemblage–a process commonly referred to as ransacking [54].

**Table 1:** Technological composition of the five archaeological assemblages from Member F (Shungura Formation) and experimental assemblage. (NB. “Cores” includes whole and broken cores, “Pebbles” includes whole and broken pebbles).

	FtJi2-A2		OMO 123 –A13		OMO 79 –A43		OMO 79 –A82			OMO 371N –A167		Exp.	
	N	%	N	%	N	%	N	%	N		%	N	%
Flakes	26	9,3	114	20,9	23	15,4	39	13,5	64		16,9	437	27,3
Broken Flakes	125	44,8	186	34,1	54	36,2	81	28,0	145		38,3	477	29,8
Flake fragments	62	22,2	132	24,2	48	32,2	94	32,5	105		27,8	181	11,3
Angular fragments	66	23,7	110	20,2	20	13,4	66	22,8	58		15,3	451	28,2
Cores	0	0	3	0,6	4	2,7	9	3,1	6		1,7	52	3,3
**TOTAL > 1 cm**	**279**	**-**	**545**	**-**	**149**	**-**	**289**	**-**	**378**		**-**	**1598**	**-**
Pebbles	8	2,9	0	0	4	2,7	0	0	3		0,6	-	-
Chips (≤1 cm)	77	21,2	423	43,7	93	37,8	29	9,1	124		24,6	-	-
**TOTAL**	**364**	**-**	**968**	**-**	**246**	**-**	**319**	**-**	**505**		**-**	**-**	**-**

### Experimental dataset analysis

The replication of stone-tool assemblages for addressing issues related to quartz and/or bipolar knapping [18, 25, 27, 28, 35, 36, 47–51] provide informative empirical datasets for establishing unicausal relationships between technical choices and the specific features of knapping products. Multi-causal relationships are, however, much more difficult to test and require joint multivariate statistics. Our approach uses context specific experimental data which are a prerequisite for technological studies heavily influenced by contextual data, especially when dealing with raw materials such as quartz, which are highly variable between contexts. In order to limit the inherent biases induced by comparative actualistic studies carried out by modern stone knappers with cognitive capacities and goals that are far removed from those of the Plio-Pleistocene hominins [55, 56], our experimental data was generated by knappers with contrasting skills.

The experimental assemblage was produced during two working sessions, in the field in 2010 and in France (Pôle Mixte de Recherche de Campagne) in 2011. They involved four novice knappers (i.e. experimenters who had never practiced stone knapping before), and one experienced knapper (MB), who routinely reproduce stone tool technologies for over twenty years and has mastered all Early Stone Age technologies. The novices were given basic training before they started, including the mechanical constraints and principles for the detachment of flakes using both bipolar and free-hand techniques. Each pebble was reduced using a single technique, either bipolar or free-hand, and for the sole purpose of obtaining as many sharp-edged products as possible, until the core was exhausted. The raw materials were quartz pebbles from the Lower Omo Valley (Fig 1B), selected to be large enough for producing flakes, i.e. > 5 cm in maximum length, whatever their knapping qualities. A total of 37 quartz pebbles were knapped, resulting in 1598 products > 1cm, for an average of 43 products per core (min. 14, max. 86) (Table 2). The entire experimental assemblage was analyzed following the same technological and morphometric attributes as those used for the archaeological assemblages. The experimental dataset is available on Nakala (https://doi.org/10.34847/nkl.3e292r29).

**Table 2:** Experimental data: Computation of useful variables for each pebble.

Pebble ID	Number of products	Knapping skills	Raw material quality	Technique	Prop. of angular fragments	Prop. of sharp-edged flakes	cutting edge length / circum.
5	55	Expert	Med_Low	Free-hand	0.273	0.342	0.507
6	42	Expert	Good	Free-hand	0.19	0.375	0.522
7	75	Novice	Good	Free-hand	0.173	0.458	0.455
8	32	Novice	Good	Free-hand	0.219	0.542	0.49
10	28	Novice	Good	Free-hand	0.071	0.48	0.465
11	32	Novice	Good	Free-hand	0.25	0.478	0.496
12	61	Expert	Good	Free-hand	0.066	0.345	0.514
13	86	Novice	Good	Free-hand	0.314	0.316	0.454
14	37	Novice	Med_Low	Free-hand	0.108	0.375	0.415
16	36	Expert	Med_Low	Bipolar	0.194	0.25	0.371
17	22	Novice	Med_Low	Bipolar	0.273	0.462	0.207
18	44	Novice	Good	Bipolar	0.136	0.189	0.413
19	41	Expert	Med_Low	Bipolar	0.341	0.28	0.441
20	60	Novice	Good	Bipolar	0.117	0.327	0.37
21	77	Novice	Good	Bipolar	0.221	0.5	0.451
22	51	Expert	Med_Low	Bipolar	0.471	0.154	0.291
23	50	Novice	Good	Bipolar	0.4	0.241	0.537
24	48	Novice	Good	Bipolar	0.521	0.429	0.4
25	27	Expert	Good	Bipolar	0.296	0.222	0.484
26	26	Novice	Good	Free-hand	0.231	0.316	0.437
27	39	Novice	Good	Free-hand	0.179	0.29	0.484
28	32	Novice	Good	Free-hand	0.375	0.444	0.466
29	48	Novice	Good	Free-hand	0.5	0.136	0.372
30	46	Novice	Good	Free-hand	0.109	0.231	0.406
31	48	Novice	Med_Low	Bipolar	0.375	0.103	0.346
32	59	Novice	Good	Bipolar	0.458	0.323	0.546
33	52	Novice	Good	Bipolar	0.231	0.385	0.413
34	66	Novice	Med_Low	Bipolar	0.742	0.188	0.316
35	20	Expert	Good	Bipolar	0.15	0.562	0.555
36	33	Expert	Med_Low	Free-hand	0.424	0.222	0.406
37	39	Expert	Good	Free-hand	0.128	0.152	0.574
38	14	Expert	Good	Free-hand	0.071	0.455	0.518
40	26	Expert	Med_Low	Bipolar	0.308	0.412	0.448
41	31	Expert	Med_Low	Free-hand	0.29	0.048	0.579
42	49	Expert	Good	Free-hand	0.041	0.13	0.522
43	41	Expert	Med_Low	Free-hand	0.317	0.154	0.57
44	25	Expert	Good	Bipolar	0.56	0.091	0.221

The isolated influence of each of the three factors (raw material knapping quality, knapping technique, and skill level) was considered in the experimental dataset for each of the three quantitative variables described above. Since the first two variables (proportion of angular fragments, proportion of sharp-edged flakes) apply to pebbles, Wilcoxon-Mann-Whitney *U* tests were performed to assess the impact of each factor on these variables for the 37 knapping sets presented in Table 2. By contrast, the third variable (cutting edge length / circumference) is relevant at the product-level, which allowed a hierarchical linear model [56] to be built for the whole sample (n = 1598), where the response variable is the ratio length/circumference, the predictor is one of the three explanatory factors, and a random intercept is defined to take into account the pebble from which each flake was produced.

In a second step, regression trees [57], a special kind of decision tree when the response variable is quantitative, were built in order to explore the combined effects of the three explanatory factors on each variable, thus allowing for the (conditional) interaction of the three factors to be presented in easily readable and interpretable form. Regression trees are based on an algorithm of recursive partitioning of the data and aim to model the relationship between one quantitative variable and a set of predictors by defining a sequence of splits from the initial dataset. Each split (i.e., each node of the tree) essentially consists in a yes/no question relative to one of the predictors, defined in such a way that it creates two subsets of individuals which maximally differ in terms of the response variable. By iterating this process, the tree grows from its initial root (i.e. the whole sample) to terminal leaves containing homogeneous subsets of individuals. Regression trees can grow until they reach a given stopping criterion. Here, as the regression trees were mainly used as exploratory tools, they were allowed to grow as long as their nodes contained more than five cases. All statistical analyses were performed with R 4.2.1 package [58]. Full details concerning the R packages used, along with the whole R code used to produce the results of the present study, can be found as Supporting Information (S1 File).

## Results

### Identifying the bipolar technique in the Shungura assemblages

**Fig 3:**
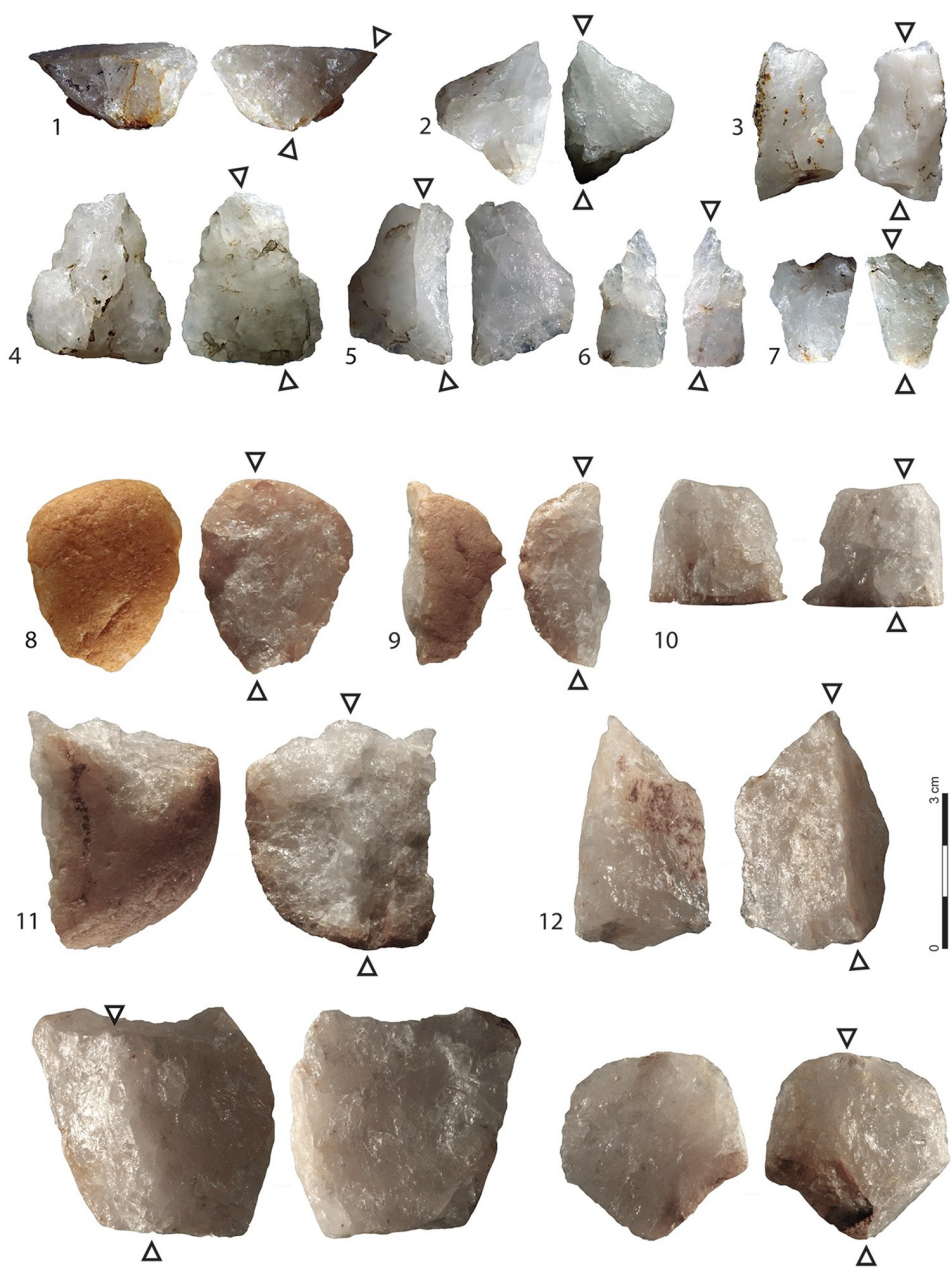
Archaeological and experimental diagnostic bipolar flakes. 1 to 7: archaeological bipolar flakes from OMO 371N –A167; 8 to 12: experimental bipolar flakes. The black triangles indicate the impact points.

The only pattern which is exclusive to the bipolar technique is the production of flakes and broken flakes with double impact points produced simultaneously during flake detachment from the struck platform, and by a counterblow from the opposite edge of the core in contact with the anvil (Fig 3). Although far from being systematic, these traits are specific to flakes obtained by bipolar percussion. When comparing their mean frequency with that observed in the five main lithic assemblages of Member F in the Shungura Formation, the results are strongly contrasted (Table 3). In two assemblages (OMO 371 –A167, OMO 79—A43), the diagnostic products appear in similar or even higher proportions than those observed in the experimental bipolar series, suggesting a predominant if not exclusive use of the bipolar technique. In two other assemblages (FtJi2 –A2, OMO 123k –A13), the proportions of diagnostic flakes are significantly lower (p < 0.001 at Fisher exact test in both cases), which can be attributed to the predominant use of the free-hand technique, while the last series (OMO 79 –A82) has an intermediate value which may reflect a more balanced mix of both techniques.

**Table 3:** Numbers and proportions of diagnostic bipolar flakes in the archaeological and experimental assemblages. (NB. only whole flakes and broken flakes preserving both their proximal and distal extremities are considered).

	FtJi2-A2	OMO123-A13	OMO79-A43	OMO79-A82	OMO371N-A167	Exp.
**N diag.**	1	8	9	4	18	32
**% diag.**	0,7	1,9	11,7	3,4	8,7	8,3
**N Total**	151	432	77	119	207	387

These strong contrasts in the use of the bipolar technique undermine the idea that the Shungura toolmakers did not have any other technical choice to reduce the rounded quartz pebbles in the Lower Omo Valley, as suggested by Ludwig [18]. Furthermore, the quartz pebbles in the Shungura Formation are not highly rounded as a result of prolonged water transport but are in the form of angular pebbles with abraded edges (Fig 1), which can be easily flaked without an initial stage of bipolar splitting for producing suitable platform angles. Nonetheless, the small size of the quartz pebbles available in the Lower Omo Valley [26] might have favored the bipolar technique as it does not require a full hand grip like the free-hand technique. The production of flakes with parallel lateral edges that are more elongated than those produced with the free-hand technique may have also been one of the advantages of the bipolar technique. By contrast, the greater loss of mass typical of the bipolar technique can be seen as a disadvantage in this context of poor mineral resources [19, 26]. Finally, during our experiments, novice knappers did not have any particular difficulty with either technique, suggesting that skill was not a critical factor in the use of the bipolar technique.

### Assessing the relative impact of raw material quality, knapping techniques and knapper skills

In order to assess the relative impact of raw material quality, knapping technique and skill levels on the selected variables, each factor was successively explored via descriptive statistics, complemented by the information provided by regression trees.

#### Proportion of angular fragments in the experimental series

**Fig 4:**
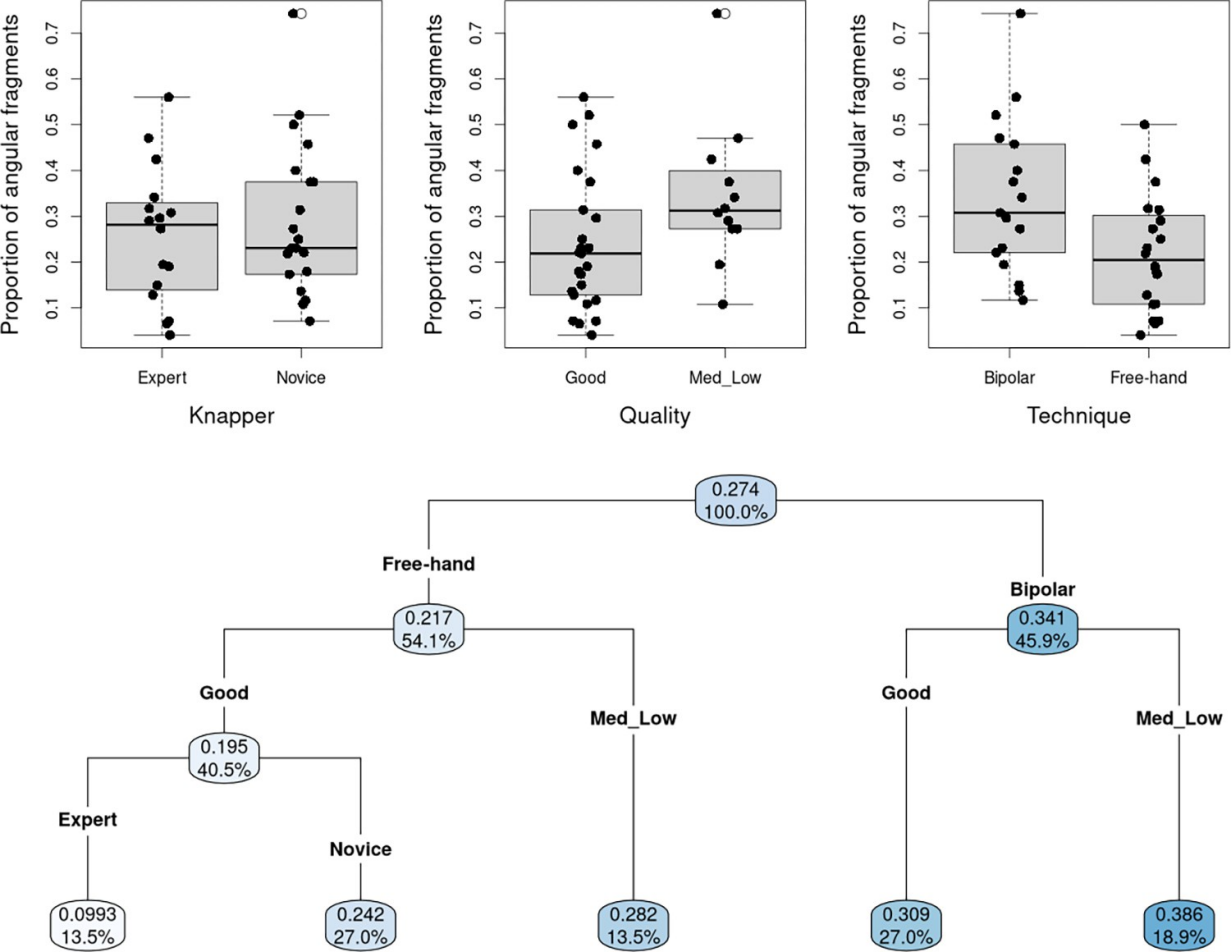
a. Boxplots and stripcharts for the impact of each factor on the proportion of angular fragments; b. Regression tree for the proportion of angular fragments, with knapping technique, raw material quality and knapping expertise as covariates. (In each node, the upper value is the empirical proportion observed for a given combination of factors; the "%" value indicates the percentage of the total sample size in this node).

The boxplots presented in Fig 4A show the proportion of angular fragments to be substantially higher for the low-quality pebbles, or when using a bipolar flaking technique. Wilcoxon-Mann-Whitney’s *U* tests provide some evidence against the null hypotheses of no impact of raw material quality and knapping technique on the proportion of angular fragments (*p* ≈ 0.051 and *p* ≈ 0.021 respectively). Conversely, knapping skill appears to have no influence on the proportion of angular fragments (*p* ≈ 0.771). Accordingly, percussion technique appears to be the most discriminating factor for the proportion of angular fragments in the regression tree (Fig 4B), with the quality of the raw material playing a consistent secondary role regardless of the technique used. Not surprisingly, low-quality pebbles are associated with a substantial increase in the proportion of angular fragments. Combining the bipolar technique with the use of low to medium-quality pebbles results in even higher rate of angular fragments (about 39%). Conversely, the free-hand technique on high-quality pebbles produces the lowest proportion of angular fragments, this trend being even more pronounced when performed by the experienced knapper (about 10%). Expertise therefore only plays a role when combined with the other two factors (free-hand technique and high-quality pebble).

#### Proportion of sharp-edged flakes

**Fig 5:**
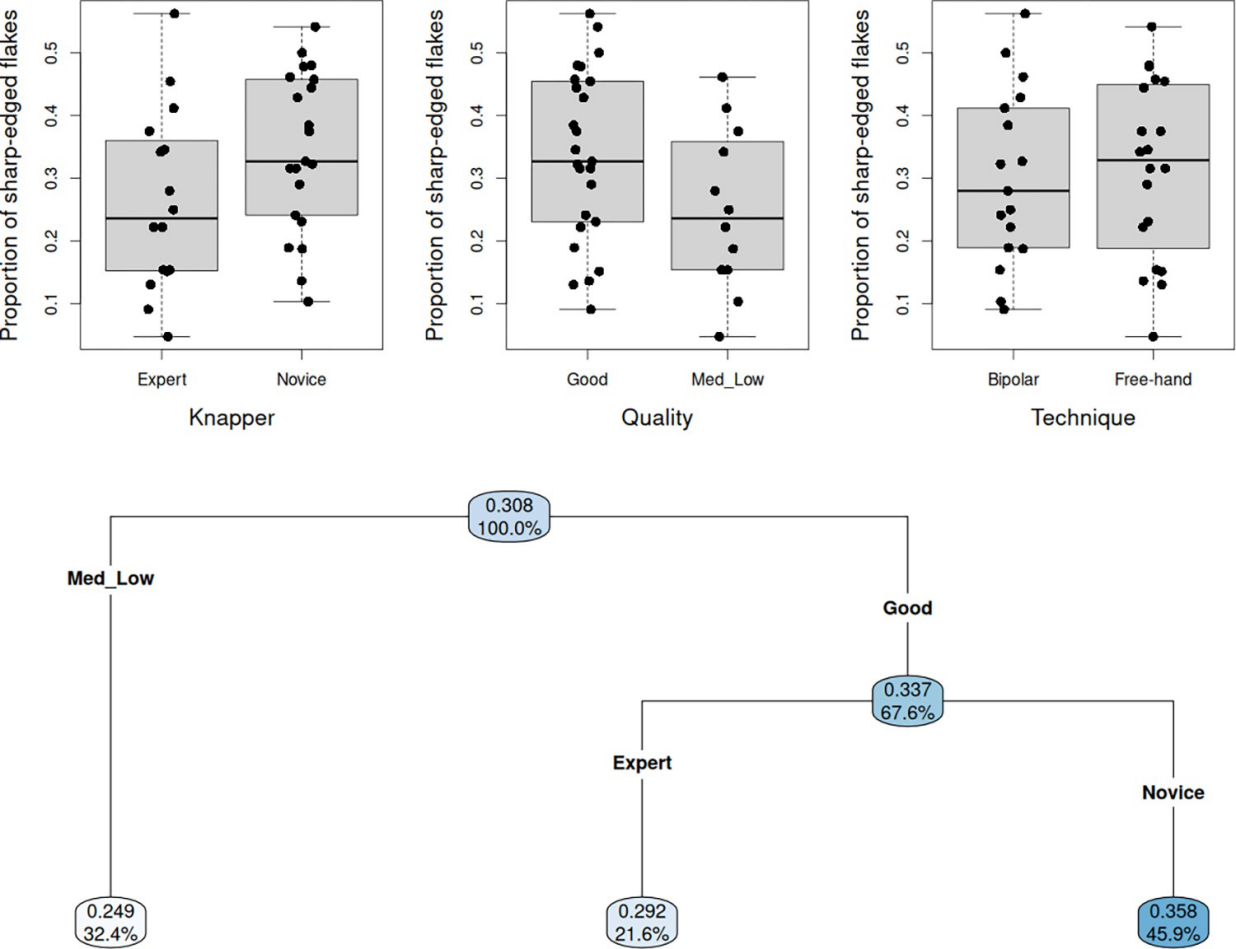
a. Boxplots and stripcharts for the impact of each factor on the proportion of sharp-edged flakes; b. Regression tree for the proportion of sharp-edged flakes, with knapping technique, raw material quality and knapping expertise as covariates. (In each node, the upper value is the empirical proportion observed for a given combination of experimental conditions; the "%" value indicates the percentage of the total sample size in this node).

As regards the proportion of sharp-edged flakes, the boxplots (Fig 5A) show quite similar outcomes for both knapping techniques. On the other hand, slightly higher proportions can be seen in the case of high-quality quartz pebbles, and among flakes produced by novice knappers. However, Wilcoxon-Mann-Whitney’s *U* tests did not provide any strong evidence against the null hypotheses of no influence of these factors (*p* ≈ 0.095 and *p* ≈ 0.075 respectively). The predominant influence of raw material quality on the proportion of sharp-edged flakes is also illustrated by the regression tree (Fig 5B), where knapper skill has an impact only when associated with good quality pebbles, and novice knappers produce an unexpected slightly higher proportion of sharp-edged flakes. Knapping technique, which was found to have no overall influence, is absent from the regression tree, thus demonstrating this factor to have no detectable influence on the proportion of sharp-edged flakes, when associated with a given raw material quality or knapping expertise. This unexpected outcome in terms of knapper skill level is not supported by a Wilcoxon Mann-Whitney test (*p* ≈ 0.31). All statistical data point to a weak impact of all three factors on the proportion of flakes with cutting edges. This likely results from either insufficient sample size, in particular for the free-hand reduction sequences on low-quality pebbles, or the unpredictability inherent in quartz pebbles from the Lower Omo Valley for detaching sharp-edged flakes, independent of technique and knapper skill.

#### Extension of cutting edges

**Fig 6:**
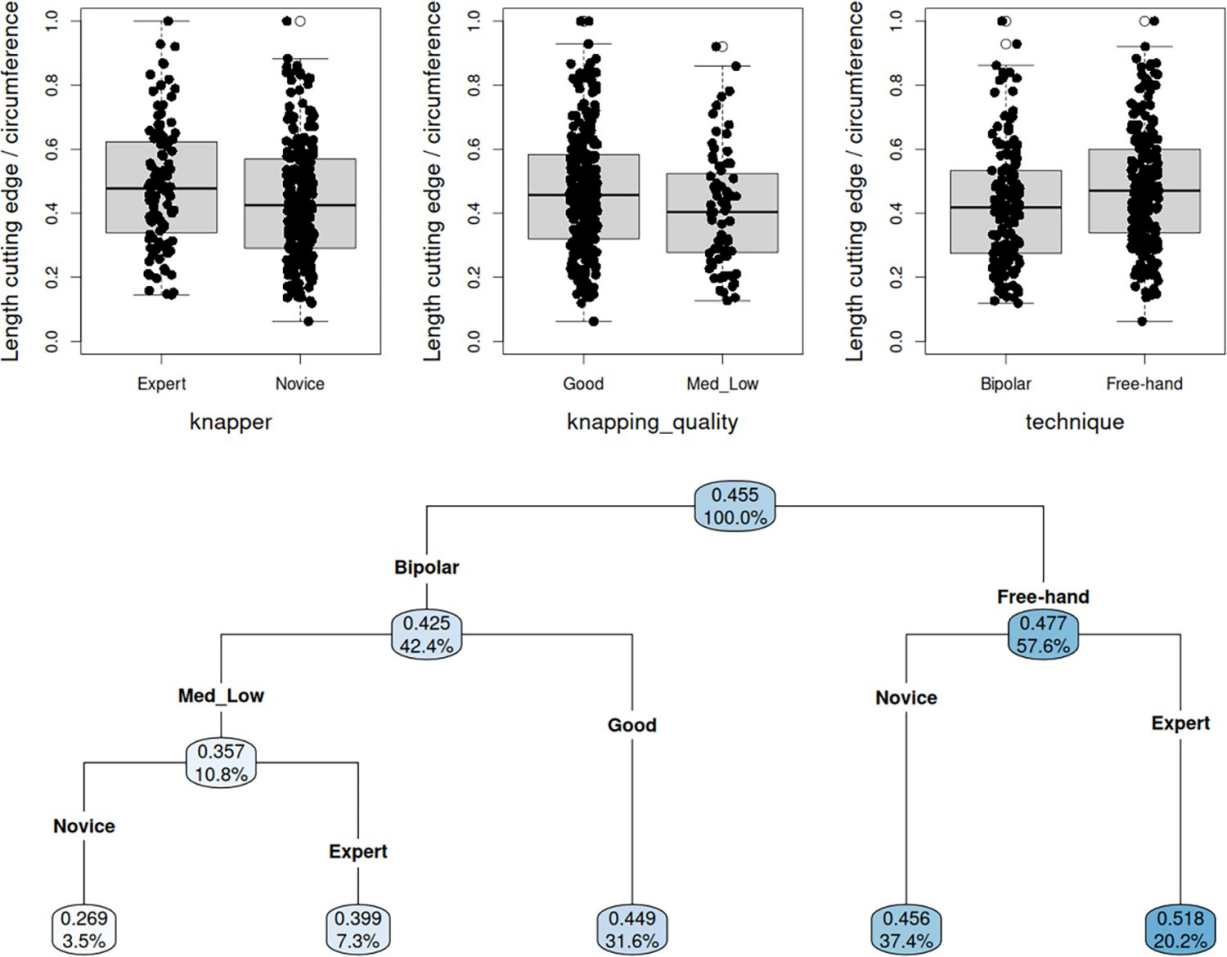
a. Boxplots and stripcharts for the impact of each factor on the extension of cutting edge; b. Regression tree for the extension of cutting edges, with knapping technique, raw material quality and knapping expertise as covariates. (In each node, the upper value is the empirical proportion observed for a given combination of experimental conditions; the "%" value indicates the percentage of the total sample size in this node).

The free-hand technique produces sharp-edged flakes and broken flakes with more extensive cutting edges, a trend which is even more evident among flakes produced from high-quality pebbles or by expert knappers (Fig 6A). Although of a moderate magnitude, these associations are confirmed by the hierarchical linear models (*p* ≈ 0.023, *p* ≈ 0.031 and *p* ≈ 0.011 respectively; see S1 File for full details). Similarly, the regression tree (Fig 6B) reflects the prevailing impact of the free-hand technique on the extension of cutting edges, which are slightly increased when combined with higher skill level, while for the bipolar technique the best results are obtained when this technique is combined with good quality quartz pebbles, regardless knapper skill level.

## Discussion

When all variables are taken into account, knapper skill level impacts successful reduction sequences only in combination with one or both of the other factors, and always to a lesser degree. The combination free-hand technique / good quality pebbles / high skill level is most efficient in minimizing the proportion of angular fragments, while free-hand technique / high skill level is the best combination for more extensive cutting edges. The unexpectedly higher ratio of sharp-edged flakes produced by the novice knappers tends to confirm the non-decisive character of knapper experience when considered alone. By contrast, knapping technique appears to be the most determinant factor for successful reduction sequences, with a marked advantage for the free-hand over bipolar technique. This raises questions concerning the potential advantages of the bipolar technique for the Shungura toolmakers.

Unlike previous descriptions of the Shungura Member F assemblages as technologically uniform [16, 18, 19], our analyses confirm the frequent, although highly variable, use of the bipolar technique. To what extent the use of the bipolar technique reflects choices and/or technical constraints cannot be precisely established. However, we assume that size constraints played a key role in the use of this technique, following a pattern which is recurrent in all contexts and all periods of prehistory [29, 59]. These constraints might have outweighed the disadvantages of this technique, notably a reduced efficiency for the production of cutting edges and a significant loss of mass, as shown by our experimental data.

Given that the bipolar technique is often found in association with the free-hand technique in the Shungura assemblages, and sometimes even in lower proportions, the bipolar technique may have been an alternative or complement to the free-hand technique. The most parsimonious explanation is that bipolar percussion was used for quartz cores that were too small to be struck in the hand, either from the beginning of the knapping sequence or at an advanced stage of core reduction, as evidenced in other contexts [60]. Technique and raw material are two highly interdependent factors in this context, with the choice of percussion technique appearing as an adaptive response to the nature of the raw material. This interpretation is consistent with the idea that raw material would be a key factor in the diversity of the Oldowan assemblages at both inter- and intra-assemblage levels [1, 19, 61, 62].

Raw material knapping quality is an aspect largely neglected in studies dedicated to the characterization of Oldowan raw materials, which mainly focus on raw material sourcing and selection [63–68], with a few exceptions (see for instance [69]). This factor deserves to be explored in greater depth, beyond the simple categorization of raw material knapping qualities applied in our study—which raw material properties were best adapted to knapping objectives, to what extent raw material quality was understood by Oldowan toolmakers and integrated in their procurement strategies are still pending issues.

The influence of raw material and/or technique overshadows the relative impact of knapper skill level. This low visibility is due to the limited technical skills required for producing sharp-edged products following a simple core-flake technology without any structured method. Minimal oral instruction coupled with a very basic demonstration were sufficient for the novice knappers involved in our experiments to fulfill the defined objectives almost as well as the experienced knapper. It has also been stressed that not all raw materials have the same potential for identifying knapper skill levels [62]. This pattern challenges the most commonly used quantitative attributes for assessing early hominin technical skills, e.g. size, proportions of end-products vs. waste products, fragmentation rates, cutting edge extension.

The low potential of the Shungura assemblages for assessing knapper technical skills should thus be seen as the combined effect of raw material constraints, a frequent use of the bipolar technique and basic technical objectives. Therefore, direct comparisons derived from technological data between the Shungura assemblages and pene-contemporaneous Oldowan assemblages are unlikely to reveal distinct skill levels. This does not mean that such differences did not exist, but rather that they are impossible to identify. This contrast is particularly evident with Lokalalei 2c, which is only about 100 km to the south in the Nachukui Formation and characterized by the controlled detachment of flakes by means of a unifacial free-hand flaking method [12, 67, 70]. It remains possible, although indemonstrable, that the same human groups were responsible for these very unequally elaborated lithic productions. Like all other early Oldowan industries, the Shungura assemblages are based on targeted knapping sequences with the goal of producing sharp-edged flakes for processing soft materials. The Shungura toolmakers managed to produce usable flakes despite the difficulties inherent to the quartz material. In agreement with de la Torre [16], we suggest that this reflects a cognitive stage far more advanced than that of any material culture known to date for non-human primates.

The mechanisms underlying the diversity of the early Oldowan assemblages therefore appear to be more linked to the cognitive skills of the toolmakers, cognitive skills which we dissociate from the technical skills considered in this study. These include a set of techno-economic behaviors based on environmental knowledge, including patterns of land-use, group mobility, raw material procurement and processing, problem-solving abilities and tool-using activities. Such aspects have remain under-explored in the Shungura Formation [26, 71] and are likely promising avenues for future research. The stable environmental conditions prevailing during the formation of Members F and Lower G [17, 72], i.e. from ca. 2.3 to 2.0 Ma, present an ideal context for inter-site diachronic comparisons in the Shungura Formation. Comparative analyses with younger Oldowan assemblages from the Fejej Formation [73], potentially allow regional patterns of change to be explored, as both contexts share the common use of vein quartz from the Hamar Range.

Several conclusions can be drawn from our analyses of the Shungura early Oldowan archaeological and experimental assemblages.

- The combination of descriptive statistics and regression trees provides a powerful tool for disentangling the relative impact of the main factors influencing the nature and composition of prehistoric lithic technologies. Applied here to the question of the factors involved in the diversity of the early Oldowan, this approach might be highly relevant in all studies involving multi-causal relationships;
- The sensorimotor and operative skills reflected in the early Oldowan assemblages from the Shungura Formation cannot be directly assessed based on standard quantitative proxies, which are highly raw material and technique dependent. This, of course, directly questions most implicit or explicit assumptions suggested in previous works regarding the skill level of the Shungura toolmakers;
- The specific environmental constraints (i.e. poor and small-sized mineral resources) of the Lower Omo Valley heavily influence the technical elaboration of the Shungura assemblages. The considerable impact of these environmental settings suggests that patterns of diversity in hominin behavioral skills are better sought in landscape knowledge and use.

## Supporting information

S1 File R code and statistical analyses. (PDF)
