# Supplementary material for: Exploring the relative influence of raw materials, percussion techniques, and hominin skill levels on the diversity of the early Oldowan assemblages: Insights from the Shungura Formation, Lower Omo Valley, Ethiopia
Source: PLoS One. 2023 Apr 5;18(4):e0283250. doi: 10.1371/journal.pone.0283250 (PMC10075482; doi:10.1371/journal.pone.0283250)
Supplement: S1 File — (PDF) [file pone.0283250.s001.pdf]

Exploring the influence of raw material properties for the identification of early hominin skill levels

Supporting Information: R code and statistical analyses

Anne Delagnes, Michel Brenet, Brad Gravina, Frédéric Santos

February 9, 2023

Contents

1 Configuration **2**

2 Load data from Nakala **3**

3 Construction of the dataframes **4**
3.1 Pebble-level: computation of useful variables (Table 2 from main text) 4
3.2 Flake-level: dataframe for linear mixed effects models 5

4 Diagnostic bipolar flakes **7**

5 Proportion of angular fragments **8**
5.1 Boxplots 8
5.2 Regression tree 8
5.3 Summary table 9
5.4 Statistical inference 10

6 Proportion of sharp-edged flakes **13**
6.1 Boxplots 13
6.2 Regression tree 13
6.3 Summary table 14
6.4 Statistical inference 14

7 Extension of cutting edges **16**
7.1 Boxplots 16
7.2 Regression tree 16
7.3 Summary table 17
7.4 Linear mixed effects models 17

1. Configuration

This document aims to facilitate the replication of a subset of results (tables or figures) presented in our article. As stated in the main text of the article, our data are hosted on Nakala (Delagnes, Brenet, Gravina, & Santos, 2022). All the analyses were performed using R (R Core Team, 2022), and this document has been built with Org mode 9.6.1 for GNU Emacs 28.2 (Schulte, Davison, Dye, & Dominik, 2012).

Along with R version 4.2.2 (2022-10-31) itself, the following R packages are loaded, using their version available on CRAN at a fixed date (2022-11-01), using the `{groundhog}` R package (Simonsohn & Gruson, 2021):

```r
## Use groundhog to improve reproducibility:
library(groundhog)

## Load the following R packages:
pkg <- c("car", "dplyr", "forcats", "janitor", "lme4",
         "rio", "rpart", "rpart.plot", "textshape")
groundhog.library(pkg = pkg, date = "2022-11-01")
```

Additional details about the R session:

```r
print(sessionInfo(), locale = FALSE)
```

```
R version 4.2.2 (2022-10-31)
Platform: x86_64-pc-linux-gnu (64-bit)
Running under: Manjaro Linux

Matrix products: default
BLAS:   /usr/lib/libopenblasp-r0.3.21.so
LAPACK: /usr/lib/liblapack.so.3.11.0

attached base packages:
[1] stats     graphics  grDevices utils     datasets  methods   base

other attached packages:
 [1] textshape_1.7.3  rpart.plot_3.1.1 rpart_4.1.19     rio_0.5.29
 [5] lme4_1.1-30      Matrix_1.5-1     janitor_2.1.0    forcats_0.5.2
 [9] dplyr_1.0.10     car_3.1-1        carData_3.0-5    groundhog_2.2.0
```

For all technical questions about this document or the R scripts included hereafter, or for any issue in reproducing the results, feel free to send an email to frederic.santos@u-bordeaux.fr.

2. Load data from Nakala

We first load the data file from Nakala, and do some basic operations of data cleaning:

```
## Load and clean the Excel sheet:
prod <- import(
    file = "https://page.hn/7qzie9",
    format = "xls",
    which = 1,
    na = c("", "-")
) |>
    mutate_at("ID expe", as.factor) |>
    select("ID expe", "Product type", "Circumference",
           "Length cutting edges", "Knapping quality",
           "Knapper", "Technique") |>
    mutate_if(is.character, as.factor) |>
    as.data.frame() |>
    clean_names() |>
    filter(!is.na(id_expe)) |>
    mutate_at(.vars = c("circumference", "length_cutting_edges"),
              .funs = function(x) as.numeric(as.character(x))) |>
    droplevels()

## Recode factor knapping_quality:
prod$knapping_quality <- fct_recode(
    prod$knapping_quality,
    Good = "GOOD",
    Med_Low = "MEDIUM to LOW"
)
```

We then display a summary of the cleaned dataframe:

```
## Display a summary:
summary(prod)
```

```
   id_expe      product_type circumference    length_cutting_edges
13      :  86   BF      :477   Min.   : 42.00   Min.   : 0.00
21      :  77   AF      :451   1st Qu.: 62.75   1st Qu.:20.00
7       :  75   F       :437   Median : 72.00   Median :31.00
34      :  66   FF      :181   Mean   : 76.83   Mean   :31.79
12      :  61   BC      : 25   3rd Qu.: 88.00   3rd Qu.:44.25
20      :  60   PC      : 10   Max.   :163.00   Max.   :95.00
(Other):1173   (Other): 17   NA's   :1226     NA's   :1226
knapping_quality   knapper          technique
Good   :1111     Expert: 591    Bipolar  :752
Med_Low: 487     Novice:1007    Free-hand:846
```

3. Construction of the dataframes

3.1. Pebble-level: computation of useful variables (Table 2 from main text)

The R code below can be used to replicate Table 2 from our main text. For each of the 37 quartz pebbles which have been knapped, three variables are computed (proportion of angular fragments, proportion of sharp-edged flakes, and extension of cutting edges as a ratio length/circumference); see main text for full details.

```
### TABLE 2 FROM MAIN TEXT.
## First compute Prop_AF variable:
bloc <- prod |>
    group_by(Expe = id_expe) |>
    summarise(
        Nb_prod = n(),
        Knapper = unique(knapper),
        Quality = unique(knapping_quality),
        Technique = unique(technique),
        Prop_AF = sum(product_type == "AF") / n())
## Then add Prop_CutEdge variable:
bloc <- data.frame(
    bloc,
    prod |>
    group_by(Expe = id_expe) |>
    subset(product_type %in% c("F", "BF", "FF")) |>
    summarise(
        Prop_CutEdge = 1 / n() * sum(!is.na(length_cutting_edges) &
                             length_cutting_edges > 0)) |>
    select(Prop_CutEdge))
## And finally, add L_Circ variable:
bloc <- data.frame(
    bloc,
    prod |>
    subset(!is.na(length_cutting_edges) & (length_cutting_edges > 0)) |>
    group_by(Expe = id_expe) |>
    summarise(L_circ = mean(length_cutting_edges / circumference)) |>
    select(L_circ))
## Display summary table:
mutate_if(bloc, is.numeric, round, 3)
```

Expe	Nb_prod	Knapper	Quality	Technique	Prop_AF	Prop_CutEdge	L_circ
5	55	Expert	Med_Low	Free-hand	0.273	0.342	0.507
6	42	Expert	Good	Free-hand	0.19	0.375	0.522
7	75	Novice	Good	Free-hand	0.173	0.458	0.455

Continued on next page

Continued from previous page

Expe	Nb_prod	Knapper	Quality	Technique	Prop_AF	Prop_CutEdge	L_circ
8	32	Novice	Good	Free-hand	0.219	0.542	0.49
10	28	Novice	Good	Free-hand	0.071	0.48	0.465
11	32	Novice	Good	Free-hand	0.25	0.478	0.496
12	61	Expert	Good	Free-hand	0.066	0.345	0.514
13	86	Novice	Good	Free-hand	0.314	0.316	0.454
14	37	Novice	Med_Low	Free-hand	0.108	0.375	0.415
16	36	Expert	Med_Low	Bipolar	0.194	0.25	0.371
17	22	Novice	Med_Low	Bipolar	0.273	0.462	0.207
18	44	Novice	Good	Bipolar	0.136	0.189	0.413
19	41	Expert	Med_Low	Bipolar	0.341	0.28	0.441
20	60	Novice	Good	Bipolar	0.117	0.327	0.37
21	77	Novice	Good	Bipolar	0.221	0.5	0.451
22	51	Expert	Med_Low	Bipolar	0.471	0.154	0.291
23	50	Novice	Good	Bipolar	0.4	0.241	0.537
24	48	Novice	Good	Bipolar	0.521	0.429	0.4
25	27	Expert	Good	Bipolar	0.296	0.222	0.484
26	26	Novice	Good	Free-hand	0.231	0.316	0.437
27	39	Novice	Good	Free-hand	0.179	0.29	0.484
28	32	Novice	Good	Free-hand	0.375	0.444	0.466
29	48	Novice	Good	Free-hand	0.5	0.136	0.372
30	46	Novice	Good	Free-hand	0.109	0.231	0.406
31	48	Novice	Med_Low	Bipolar	0.375	0.103	0.346
32	59	Novice	Good	Bipolar	0.458	0.323	0.546
33	52	Novice	Good	Bipolar	0.231	0.385	0.413
34	66	Novice	Med_Low	Bipolar	0.742	0.188	0.316
35	20	Expert	Good	Bipolar	0.15	0.562	0.555
36	33	Expert	Med_Low	Free-hand	0.424	0.222	0.406
37	39	Expert	Good	Free-hand	0.128	0.152	0.574
38	14	Expert	Good	Free-hand	0.071	0.455	0.518
40	26	Expert	Med_Low	Bipolar	0.308	0.412	0.448
41	31	Expert	Med_Low	Free-hand	0.29	0.048	0.579
42	49	Expert	Good	Free-hand	0.041	0.13	0.522
43	41	Expert	Med_Low	Free-hand	0.317	0.154	0.57
44	25	Expert	Good	Bipolar	0.56	0.091	0.221

Table 2: Computation of useful variables for each pebble.

3.2. Flake-level: dataframe for linear mixed effects models

The dataframe presented in Table 2 gives data at the pebble-level. Linear mixed effects models (Faraway, 2006; Gałecki & Burzykowski, 2013) can also be used to study the data at the flake/product-level. To build such models, we first have to create another version of the dataframe from the flakes/products data, and add a binary variable (`AF`) built from the factor `product_type` (i.e., `AF=yes` iff the flake is an angular fragment, `AF=no` otherwise). A similar

binary factor, CE, is created for the proportion of sharp-edged flakes. We display below the first five rows of this dataframe.

```
## Create a new version of the dataframe:
dtf <- select(prod, id_expe, product_type, technique, knapping_quality,
              knapper, circumference, length_cutting_edges)

## Add a binary factor for angular fragments:
dtf$AF <- fct_collapse(
    .f = dtf$product_type,
    yes = "AF",
    other_level = "no"
)
## Add a binary factor for sharp-edged flakes:
dtf$CE <- ifelse(
    !is.na(dtf$length_cutting_edges) & dtf$length_cutting_edges > 0,
    yes = "yes",
    no = "no"
) |> as.factor()
## Keep only those fragments that have L>0 and Circ>0:
dtf$L_circ <- ifelse(
    (dtf$length_cutting_edges > 0) & (!is.na(dtf$length_cutting_edges)),
    yes = dtf$length_cutting_edges / dtf$circumference,
    no = NA
)

## Display the first five rows:
head(dtf, 5)
```

```
  id_expe product_type technique knapping_quality knapper circumference
1       5           AF Free-hand          Med_Low  Expert            NA
2       5           AF Free-hand          Med_Low  Expert            NA
3       5           AF Free-hand          Med_Low  Expert            NA
4       5           AF Free-hand          Med_Low  Expert            NA
5       5           AF Free-hand          Med_Low  Expert            NA
  length_cutting_edges  AF  CE L_circ
1                   NA yes no     NA
2                   NA yes no     NA
3                   NA yes no     NA
4                   NA yes no     NA
5                   NA yes no     NA
```

4. Diagnostic bipolar flakes

Table 3 (in our main text and below) summarizes the data relative to the discussion about the presence and significance of the bipolar technique among archaeological assemblages, through the observed proportion of diagnostic flakes.

```
### TABLE 3:
diagn <- matrix(c(355, 32, 150, 1, 68, 9, 115, 4, 424, 8, 189, 18),
                nrow = 2)
rownames(diagn) <- c("Other", "Diagnostic")
colnames(diagn) <- c("Exp", "FtJi2-A2", "OMO79-A43",
                     "OMO79-A82", "OMO123k-A13", "OMO371N-A167")
print(diagn)
```

	Exp	FtJi2-A2	OMO79-A43	OMO79-A82	OMO123k-A13	OMO371N-A167
Other	355	150	68	115	424	189
Diagnostic	32	1	9	4	8	18

Table 3: Amount of bipolar diagnostic flakes in the archaeological and experimental assemblages.

We first perform a Fisher exact test on this contingency table:

```
## Omnibus Fisher exact test:
fisher.test(diagn, simulate.p.value = TRUE, B = 4999)
```

```

        Fisher's Exact Test for Count Data with simulated p-value (based on
        4999 replicates)

data:  diagn
p-value = 2e-04
alternative hypothesis: two.sided
```

We can further analyze the differences between the experimental assemblage and each archaeological assemblage using Fisher exact tests on the corresponding 2×2 sub-tables — a process known as *ransacking* (Sharpe, 2015). The p-values for the Fisher exact tests between the experimental assemblage and each archaeological assemblage can be obtained as follows:

```
## Ransacking:
pvals <- rep(NA, 5); names(pvals) <- colnames(diagn)[-1]
for (i in 1:5) {
    pvals[i] <- fisher.test(diagn[, c(1, i+1)])$p.value
}
round(pvals, 4)
```

```
FtJi2-A2    OMO79-A43    OMO79-A82   OMO123k-A13 OMO371N-A167
  0.0004       0.3773       0.1002        0.0000       0.8774
```

5. Proportion of angular fragments

5.1. Boxplots

We first define a custom function/helper for the boxplots presented in our article:

```
my_bxp <- function(formula, data,
                   ylab = "Proportion of angular fragments", ...)
{
    boxplot(formula, data = data, ylab = ylab, ...)
    stripchart(formula, data = data, vertical = TRUE,
               add = TRUE, pch = 16, method = "jitter")
}
```

Then, the boxplots presented in Figure 4 of our main text were produced using the following R code:

```
### FIGURE 4 FROM MAIN TEXT (boxplots).
## Graphical comparison of Prop_AF for each factor:
par(mfrow = c(1, 3), cex = 1.3, cex.lab = 1.35)
my_bxp(Prop_AF ~ Knapper, data = bloc)
my_bxp(Prop_AF ~ Quality, data = bloc)
my_bxp(Prop_AF ~ Technique, data = bloc)
```

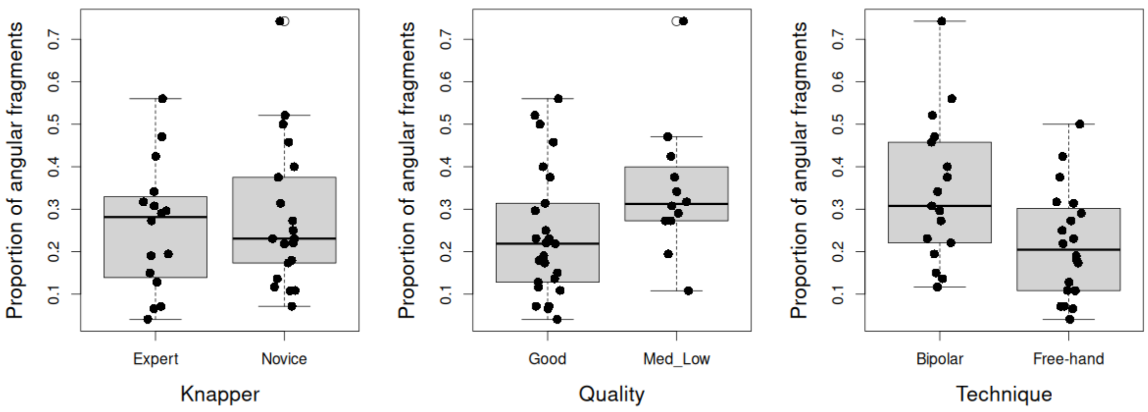

5.2. Regression tree

The regression tree presented in Figure 4 of our main text was produced using the R code below.

```
### FIGURE 4 FROM MAIN TEXT (regression tree).
## Build a decision tree for angular fragments:
arbre_af <- rpart(Prop_AF ~ Knapper + Quality + Technique,
                  data = bloc, minbucket = 5)
```

```r
rpart.plot(
    x = arbre_af,
    cex = 1.5,
    type = 4,
    clip.facs = TRUE,
    digits = 3
)
```

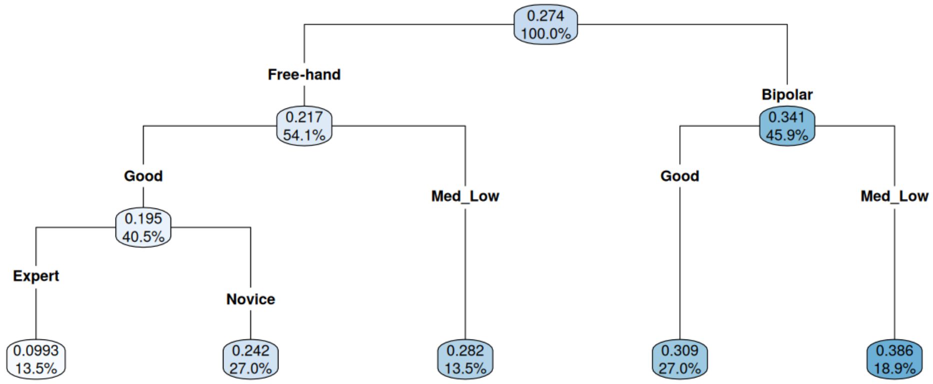

5.3. Summary table

Table 4 below complements the regression tree in Figure 4.

```r
### SUMMARY TABLE (ANGULAR FRAG.):
bloc |>
    group_by(Technique, Quality, Knapper) |>
    summarise(
        n = n(),
        Prop_AF = round(mean(Prop_AF), 3)
    ) |>
    as.data.frame()
```

Technique	Quality	Knapper	n	Prop_AF
Bipolar	Good	Expert	3	0.335
Bipolar	Good	Novice	7	0.298
Bipolar	Med_Low	Expert	4	0.329
Bipolar	Med_Low	Novice	3	0.463
Free-hand	Good	Expert	5	0.099
Free-hand	Good	Novice	10	0.242
Free-hand	Med_Low	Expert	4	0.326
Free-hand	Med_Low	Novice	1	0.108

Table 4: Average proportion of angular fragments for each possible combination of technique, quality and technique.

5.4. Statistical inference

5.4.1. Wilcoxon tests

We first perform a Wilcoxon text for the proportion of angular fragments depending on raw material quality, technique and knapper expertise respectively.

```
## Wilcoxon test for AF by quality:
wilcox.test(Prop_AF ~ Quality, data = bloc)
```

```

	Wilcoxon rank sum test with continuity correction

data:  Prop_AF by Quality
W = 89.5, p-value = 0.05152
alternative hypothesis: true location shift is not equal to 0
```

```
## Wilcoxon test for AF by expertise:
wilcox.test(Prop_AF ~ Knapper, data = bloc)
```

```

	Wilcoxon rank sum test with continuity correction

data:  Prop_AF by Knapper
W = 158, p-value = 0.7708
alternative hypothesis: true location shift is not equal to 0
```

```
## Wilcoxon test for AF by technique:
wilcox.test(Prop_AF ~ Technique, data = bloc)
```

```

	Wilcoxon rank sum test with continuity correction

data:  Prop_AF by Technique
W = 246.5, p-value = 0.02052
alternative hypothesis: true location shift is not equal to 0
```

5.4.2. Generalized linear mixed effects models

The following generalized linear mixed effect model (not presented in the main text) explains the binary factor `AF` by the technique used, setting a random intercept depending on the pebble ID.

```
## Build a GLMM for angular fragments by technique:
mod_af_tech <- glmer(AF ~ technique + (1|id_expe),
                     family = binomial, data = dtf)
summary(mod_af_tech)
```

```
Generalized linear mixed model fit by maximum likelihood (Laplace
  Approximation) [glmerMod]
 Family: binomial  ( logit )
Formula: AF ~ technique + (1 | id_expe)
   Data: dtf

     AIC      BIC   logLik deviance df.resid
  1781.1   1797.2   -887.5   1775.1     1595

Scaled residuals:
    Min      1Q  Median      3Q     Max
-3.3284 -0.9982  0.4638  0.6290  1.5081

Random effects:
 Groups  Name        Variance Std.Dev.
 id_expe (Intercept) 0.4874   0.6981
Number of obs: 1598, groups:  id_expe, 37

Fixed effects:
                   Estimate Std. Error z value Pr(>|z|)
(Intercept)          0.7167     0.1906   3.759  0.00017 ***
techniqueFree-hand   0.6858     0.2638   2.600  0.00933 **
---
Signif. codes:  0 '***' 0.001 '**' 0.01 '*' 0.05 '.' 0.1 ' ' 1

Correlation of Fixed Effects:
            (Intr)
tchnqFr-hnd -0.719
```

The analysis of deviance table can be obtained as follows:

```
Anova(mod_af_tech)
```

```
Analysis of Deviance Table (Type II Wald chisquare tests)

Response: AF
           Chisq Df Pr(>Chisq)
technique 6.7578  1   0.009334 **
---
Signif. codes:  0 '***' 0.001 '**' 0.01 '*' 0.05 '.' 0.1 ' ' 1
```

Similar analyses can be performed replacing the explanatory factor "technique" by the raw material quality:

```
## Build a GLMM for angular fragments by quality:
mod_af_qlt <- glmer(AF ~ knapping_quality + (1|id_expe),
                    family = binomial, data = dtf)
Anova(mod_af_qlt)
```

```
Analysis of Deviance Table (Type II Wald chisquare tests)
```

```
Response: AF
                  Chisq Df Pr(>Chisq)
knapping_quality 4.2802  1    0.03856 *
---
Signif. codes:  0 '***' 0.001 '**' 0.01 '*' 0.05 '.' 0.1 ' ' 1
```

or by the knapper experience:

```r
## Build a GLMM for angular fragments by knapping experience:
mod_af_exp <- glmer(AF ~ knapper + (1|id_expe),
                    family = binomial, data = dtf)
Anova(mod_af_exp)
```

```
Analysis of Deviance Table (Type II Wald chisquare tests)

Response: AF
         Chisq Df Pr(>Chisq)
knapper 0.3779  1     0.5387
```

6. Proportion of sharp-edged flakes

6.1. Boxplots

Then, the boxplots presented in Figure 5 of our main text were produced using the following R code:

```
### FIGURE 5 FROM MAIN TEXT (boxplots):
par(mfrow = c(1, 3), cex = 1.3, cex.lab = 1.35)
my_bxp(Prop_CutEdge ~ Knapper, data = bloc,
       ylab = "Proportion of sharp-edged flakes")
my_bxp(Prop_CutEdge ~ Quality, data = bloc,
       ylab = "Proportion of sharp-edged flakes")
my_bxp(Prop_CutEdge ~ Technique, data = bloc,
       ylab = "Proportion of sharp-edged flakes")
```

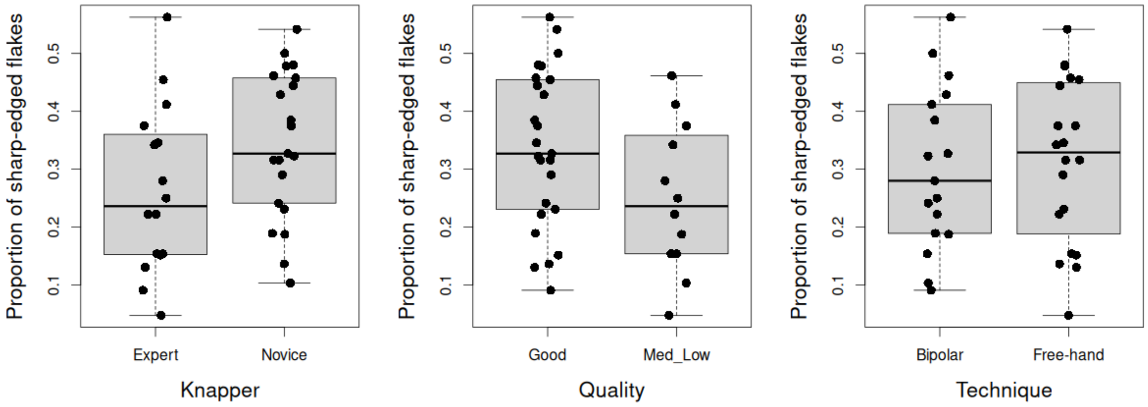

6.2. Regression tree

The regression tree presented in Figure 5 of our main text was produced using the R code below.

```
### FIGURE 5 FROM MAIN TEXT (regression tree).
arbre_ce <- rpart(Prop_CutEdge ~ Knapper + Quality + Technique,
                  data = bloc, minbucket = 4)
rpart.plot(
    x = arbre_ce,
    cex = 1.5,
    type = 4,
    clip.facs = TRUE,
    digits = 3
)
```

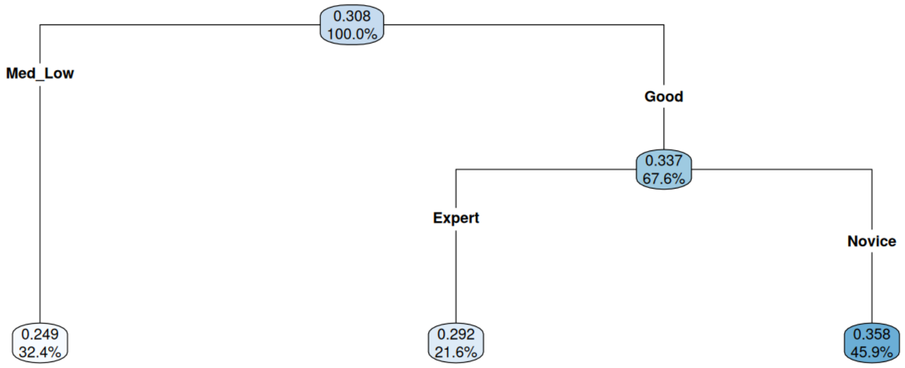

6.3. Summary table

Table 5 below complements the regression tree in Figure 5.

```
### SUMMARY TABLE:
bloc |>
    group_by(Technique, Quality, Knapper) |>
    summarise(
        n = n(),
        Prop_CutEdges = round(mean(Prop_CutEdge), 3)
    ) |>
    as.data.frame()
```

Technique	Quality	Knapper	n	Prop_CutEdges
Bipolar	Good	Expert	3	0.292
Bipolar	Good	Novice	7	0.342
Bipolar	Med_Low	Expert	4	0.274
Bipolar	Med_Low	Novice	3	0.251
Free-hand	Good	Expert	5	0.291
Free-hand	Good	Novice	10	0.369
Free-hand	Med_Low	Expert	4	0.191
Free-hand	Med_Low	Novice	1	0.375

Table 5: Average proportion of sharp-edged flakes for each possible combination of technique, quality and technique.

6.4. Statistical inference

6.4.1. Wilcoxon tests

Three Wilcoxon tests for the proportion of sharp-edged flakes (by raw material quality, knapping expertise and technique respectively) are performed below.

```r
## Wilcoxon test by quality:
wilcox.test(Prop_CutEdge ~ Quality, data = bloc)
```

```

	Wilcoxon rank sum test with continuity correction

data:  Prop_CutEdge by Quality
W = 202, p-value = 0.09467
alternative hypothesis: true location shift is not equal to 0
```

```r
## Wilcoxon test by technique:
wilcox.test(Prop_CutEdge ~ Technique, data = bloc)
```

```

	Wilcoxon rank sum test with continuity correction

data:  Prop_CutEdge by Technique
W = 161, p-value = 0.7956
alternative hypothesis: true location shift is not equal to 0
```

```r
## Wilcoxon test by knapping expertise:
wilcox.test(Prop_CutEdge ~ Knapper, data = bloc)
```

```

	Wilcoxon rank sum test with continuity correction

data:  Prop_CutEdge by Knapper
W = 109.5, p-value = 0.07532
alternative hypothesis: true location shift is not equal to 0
```

6.4.2. Generalzed linear mixed effects models

A generalized linear mixed effects model for the presence of sharp-edged flakes depending on the technique used, setting a random intercept depending on the pebble ID, is defined below.

```r
## Build a GLMM for proportion of sharp-edged flakes by technique:
mod_ce_tech <- glmer(CE ~ technique + (1|id_expe),
                     family = binomial, data = dtf)
Anova(mod_ce_tech)
```

```
Analysis of Deviance Table (Type II Wald chisquare tests)

Response: CE
           Chisq Df Pr(>Chisq)
technique 1.3193  1     0.2507
```

Similar models can be built depending of raw material quality or knapping expertise (extensive results not shown here).

7. Extension of cutting edges

7.1. Boxplots

The boxplots presented in Figure 6 of our main text were produced using the following R code:

```
### FIGURE 6 FROM MAIN TEXT (boxplots):
par(mfrow = c(1, 3), cex = 1.3, cex.lab = 1.35)
my_bxp(L_circ ~ knapper, data = dtf, ylim = 0:1,
       ylab = "Length cutting edge / circumference")
my_bxp(L_circ ~ knapping_quality, data = dtf, ylim = 0:1,
       ylab = "Length cutting edge / circumference")
my_bxp(L_circ ~ technique, data = dtf, ylim = 0:1,
       ylab = "Length cutting edge / circumference")
```

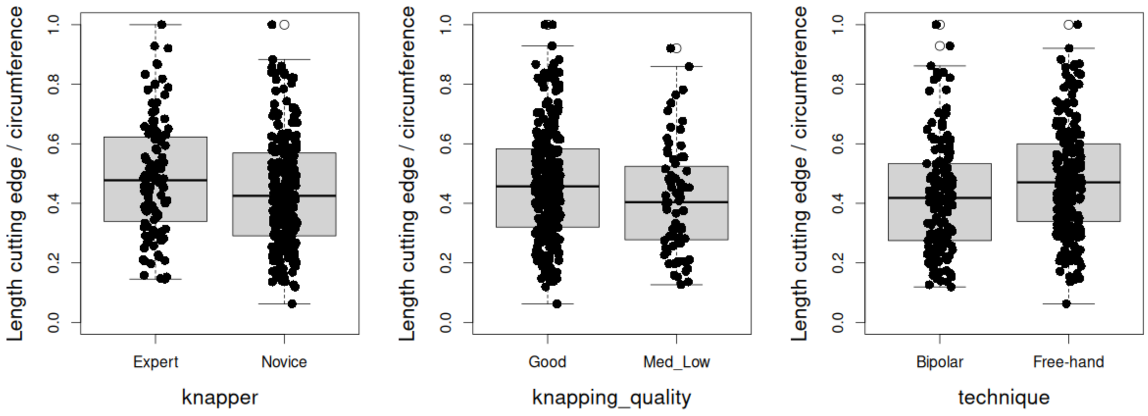

7.2. Regression tree

The regression tree presented in Figure 6 of our main text was produced using the R code below.

```
### FIGURE 6 FROM MAIN TEXT (regression tree):
arbre_lcirc <- rpart(L_circ ~ knapper + knapping_quality + technique,
                     data = dtf, minbucket = 5)
rpart.plot(
    x = arbre_lcirc,
    cex = 1.5,
    type = 4,
    clip.facs = TRUE,
    digits = 3
)
```

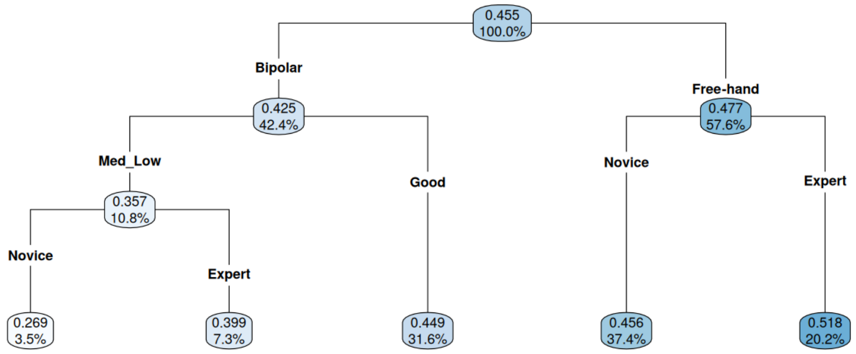

7.3. Summary table

Table 6 below complements the regression tree in Figure 6.

```
### SUMMARY TABLE:
dtf |>
    group_by(Technique = technique,
             Quality = knapping_quality,
             Knapper = knapper) |>
    summarise(
        n = n(),
        Ratio_length_circ = round(mean(L_circ, na.rm = TRUE), 3)
    ) |>
    as.data.frame()
```

Technique	Quality	Knapper	n	Ratio_length_circ
Bipolar	Good	Expert	72	0.511
Bipolar	Good	Novice	390	0.439
Bipolar	Med_Low	Expert	154	0.399
Bipolar	Med_Low	Novice	136	0.269
Free-hand	Good	Expert	205	0.524
Free-hand	Good	Novice	444	0.46
Free-hand	Med_Low	Expert	160	0.504
Free-hand	Med_Low	Novice	37	0.415

Table 6: Mean value of the ratio length of cutting edge / circumference for each possible combination of technique, quality and technique.

7.4. Linear mixed effects models

First we define a linear fixed effect model to assess the impact of knapping expertise on the ratio length/circumference:

```r
## Build a LMER for the ratio length/circumference by knapping expertise:
mod_ratio_knap <- lmer(L_circ ~ knapper + (1|id_expe), data = dtf)
Anova(mod_ratio_knap)
```

```
Analysis of Deviance Table (Type II Wald chisquare tests)

Response: L_circ
         Chisq Df Pr(>Chisq)
knapper 5.1597  1    0.02312 *
---
Signif. codes:  0 '***' 0.001 '**' 0.01 '*' 0.05 '.' 0.1 ' ' 1
```

A similar model can be built to assess the impact of the quality of raw material:

```r
## Build a LMER for the ratio length/circumference by quality of material:
mod_ratio_qlt <- lmer(L_circ ~ knapping_quality + (1|id_expe), data = dtf)
Anova(mod_ratio_qlt)
```

```
Analysis of Deviance Table (Type II Wald chisquare tests)

Response: L_circ
                  Chisq Df Pr(>Chisq)
knapping_quality 4.6224  1    0.03156 *
---
Signif. codes:  0 '***' 0.001 '**' 0.01 '*' 0.05 '.' 0.1 ' ' 1
```

And similarly for the impact of the technique used:

```r
## Build a LMER for the ratio length/circumference by technique:
mod_ratio_tech <- lmer(L_circ ~ technique + (1|id_expe), data = dtf)
Anova(mod_ratio_tech)
```

```
Analysis of Deviance Table (Type II Wald chisquare tests)

Response: L_circ
           Chisq Df Pr(>Chisq)
technique 6.4522  1    0.01108 *
---
Signif. codes:  0 '***' 0.001 '**' 0.01 '*' 0.05 '.' 0.1 ' ' 1
```

References

Delagnes, A., Brenet, M., Gravina, B., & Santos, F. (2022). *Lithic experimental dataset.* Nakala. Retrieved from https://nakala.fr/10.34847/nkl.3e292r29

Faraway, J. J. (2006). *Extending the linear model with R: Generalized linear, mixed effects and nonparametric regression models*. Boca Raton: Chapman & Hall/CRC.

Gałecki, A. T., & Burzykowski, T. (2013). *Linear mixed-effects models using R: A step-by-step approach*. New York, NY: Springer.

R Core Team. (2022). *R: A language and environment for statistical computing. R Foundation for Statistical Computing, Vienna, Austria.* Retrieved from https://www.R-project.org/

Schulte, E., Davison, D., Dye, T., & Dominik, C. (2012). A Multi-Language Computing Environment for Literate Programming and Reproducible Research. *Journal of Statistical Software*, *46*(1), 1–24. doi:10.18637/jss.v046.i03

Sharpe, D. (2015). Chi-Square Test is Statistically Significant: Now What? *Practical Assessment, Research, and Evaluation*, *20*(1). doi:10.7275/tbfa-x148

Simonsohn, U., & Gruson, H. (2021). *Groundhog: Reproducible Scripts via Version-Specific Package Loading.* Retrieved from https://CRAN.R-project.org/package=groundhog
